# Improving the large scale purification of the HIV microbicide, griffithsin

**DOI:** 10.1186/s12896-015-0120-5

**Published:** 2015-02-22

**Authors:** Joshua L Fuqua, Valentine Wanga, Kenneth E Palmer

**Affiliations:** Owensboro Cancer Research Program, 1020 Breckenridge St., Suite 201, Owensboro, KY 42303 USA; University of Louisville School of Medicine, James Graham Brown Cancer Center, 529 S Jackson Street, Louisville, KY 40202 USA; Institute for Health Metrics and Evaluation, University of Washington, 2301 5th Ave, Suite 600, Seattle, WA USA

**Keywords:** Griffithsin, Tobacco mosaic virus, Protein purification, *N. benthamiana*, Bentonite, Microbicide, HIV

## Abstract

**Background:**

Griffithsin is a broad spectrum antiviral lectin that inhibits viral entry and maturation processes through binding clusters of oligomannose glycans on viral envelope glycoproteins. An efficient, scaleable manufacturing process for griffithsin active pharmaceutical ingredient (API) is essential for particularly cost-sensitive products such as griffithsin -based topical microbicides for HIV-1 prevention in resource poor settings. Our previously published purification method used ceramic filtration followed by two chromatography steps, resulting in a protein recovery of 30%. Our objective was to develop a scalable purification method for griffithsin expressed in *Nicotiana benthamiana* plants that would increase yield, reduce production costs, and simplify manufacturing techniques. Considering the future need to transfer griffithsin manufacturing technology to resource poor areas, we chose to focus modifying the purification process, paying particular attention to introducing simple, low-cost, and scalable procedures such as use of temperature, pH, ion concentration, and filtration to enhance product recovery.

**Results:**

We achieved >99% pure griffithsin API by generating the initial green juice extract in pH 4 buffer, heating the extract to 55°C, incubating overnight with a bentonite MgCl_2_ mixture, and final purification with Capto™ multimodal chromatography. Griffithsin extracted with this protocol maintains activity comparable to griffithsin purified by the previously published method and we are able to recover a substantially higher yield: 88 ± 5% of griffithsin from the initial extract. The method was scaled to produce gram quantities of griffithsin with high yields, low endotoxin levels, and low purification costs maintained.

**Conclusions:**

The methodology developed to purify griffithsin introduces and develops multiple tools for purification of recombinant proteins from plants at an industrial scale. These tools allow for robust cost-effective production and purification of griffithsin. The methodology can be readily scaled to the bench top or industry and process components can be used for purification of additional proteins based on biophysical characteristics.

## Background

Griffithsin (GRFT) is a high mannose targeting lectin that has shown promise as a microbicide candidate against viruses with oligomannose-rich envelope glycoproteins, such as HIV-1 or HSV-2 [1-4]. GRFT was originally isolated from a red alga (*Griffithsia* sp.) with native and recombinant forms showing similar antiviral activity against laboratory strains and primary isolates of HIV-1 [1,4]. Recombinant forms of GRFT have been successfully produced in both *E. coli* and *N. benthamiana*, with the latter used to produce multi-gram quantities of functional GRFT [4,5]. GRFT has shown to be a safe and effective microbicide against HIV, but needs to be evaluated in clinical trials [2,6]. The HIV epidemic is prevalent in many resource-limited or developing countries and the availability of indigenous manufacturing systems for GRFT in those areas could facilitate large-scale rollout of microbicide product. Cost and production complexity are factors that must be considered when manufacturing biologics for extremely cost-sensitive markets, such as for microbicides in developing economies.

This communication details our development of a process to reduce the production cost of GRFT from *N. benthamiana*. Currently published industrial scale GRFT manufacturing uses ceramic filtration followed by two-stage chromatography [4]. These relatively complex methods require specialized equipment and increase production costs. The molecule’s biophysical properties, and specifically the melting temperature, were taken into account when developing a new purification methodology. GRFT has a melting temperature of 78.8°C and remains functionally stable at temperatures not tolerated by many of the contaminating proteins [7,8]. There are three primary contaminants to contend with when trying to purify GRFT from *N. benthamiana*: two production host-associated, the small and large subunits of Ribulose-1,5-bisphosphate carboxylase/oxygenase (RuBisCO), and one contaminant from the TMV production system, TMV coat protein (CP). RuBisCO lacks the thermostability of GRFT at acidic pHs. Therefore, in these studies we tested heat treatment, under acidic conditions, of our crude plant extracts containing GRFT to determine if any of the major contaminants could be removed through precipitation/denaturation. The TMV CP has shown a propensity to self-aggregate under certain ionic, pH, and temperature conditions. Absorption by the clay material, bentonite, has been previously used to purify TMV particles and could potentially be a useful tool in removal of the coat protein contaminant [9]. Therefore purification methods utilizing altered heat, pH, ion concentrations, and bentonite filtration were all tested in an effort to develop a scalable purification method requiring less specialized equipment. We have developed a single-step-chromatography purification method that will decrease the cost and complexity of producing large quantities of GRFT, while increasing the protein recovery. The application of this knowledge on industrial size purification should further reduce GRFT cost of production and may be beneficial for production of other plant-made pharmaceuticals.

## Results and discussion

**Figure 1:**
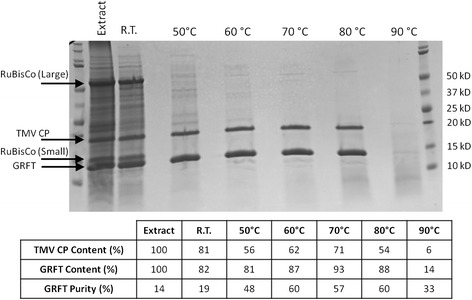
**Removal of plant protein contaminants from GRFT Extract with Heat.** Plants expressing GRFT through a TMV-based expression system were harvested at pH4 in sodium acetate buffer and the extract was divided into 7 aliquots. Each aliquot was incubated for 15 minutes at temperatures ranging from room temperature (24°C) to 90°C and centrifuged to remove any precipitant. Samples of the initial extract and all assessed temperatures were analyzed by SDS-PAGE with coomassie staining. The initial extract contains multiple contaminating proteins, which remain after incubation at room temperature. Once the extracts are heated to at least 50°C the majority of contaminating proteins precipitate except for the target protein GRFT and the TMV coat protein. The composition of the extract remains similar with temperature treatments ranging from 50 – 80°C, but at 90°C incubation nearly all proteins are precipitated. Table: Densitometry readings corresponding to SDS-PAGE shown in Figure 1.

Leaf punches were used to determine the pH and NaCl concentration parameters that were optimal for extraction of GRFT and minimal extraction of contaminating proteins. One dimensional SDS PAGE profiles showed the optimal extraction pH for GRFT to be pH 4.0. The addition of 100 mM NaCl showed minimal impact on the purity of GRFT in the small-scale samples, but from prior work it is understood that the addition of NaCl will improve purity as long as it is not deleterious to the protein of interest especially as scale is increased. Therefore all subsequent purifications used 100 mM sodium acetate and 300 mM NaCl pH4 as the extraction buffer. Analysis of the small scale protein extraction profiles by SDS-PAGE demonstrates little impact of temperature on the extract purity, but does show that GRFT maintains stability in temperatures ranging from 60 – 80°C. GRFT’s thermostability in heated extracts was further assessed in larger scale extractions and Figure 1 shows clearly the impact that heat has on contaminating proteins. Nearly all contaminating proteins aggregated at temperatures above 50°C (Figure 1), increasing the extract purity 30 – 40% (Figure 1), while GRFT remains stable until the extract reaches 90°C.

**Figure 2:**
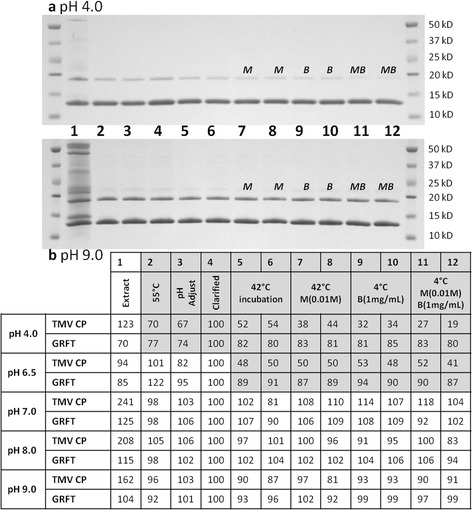
**Effects of pH on the activity of MgCl**_**2**_ **and bentonite to remove the TMV coat protein.** Extracts were harvested in sodium acetate (pH 4–5) or tris (pH7-9) buffer depending on the desired pH range and were visualized using coomassie stained SDS-PAGE. Sample 1 shows the proteins in the initial extract and sample 2 represents the proteins after the extract is heated to 55°C and centrifuged. Sample 3 represents the extract after it was adjusted to its corresponding pH **a)** pH 4.0 and **b)** pH 9.0. Sample 3 extracts were centrifuged to remove additional precipitate and represented in sample 4. Samples 5–12 represent various temperature, bentonite, or MgCl_2_ treatments of sample 4 performed in duplicate. MgCl_2_ (M), bentonite (B), and bentonite & MgCl_2_ (MB) Samples 5 & 6 were incubated overnight at 42°C. Samples 7 & 8 were incubated overnight at 42°C after treatment with 0.01 M MgCl_2_. Samples 9 & 10 were incubated overnight at 4°C with the addition of 1 mg/mL bentonite. Samples 11 & 12 were incubated overnight at 4°C after the addition of 1 mg/mL bentonite & 0.01 M MgCl_2_. Table: Densitometry readings of SDS-PAGE represented in Figure 2 with values from three additional gels representing additional pH values that were explored (pH 6.5, 7.0, 8.0). The shaded area represents an extract condition achieving greater than 80% purity of GRFT.

The primary contaminating protein left in the GRFT extract after heating was the TMV CP. Therefore, methods for removing the TMV CP were examined. These included the addition of bentonite and/or MgCl_2_, as well as pH and temperature adjustments. The more acidic extracts pH 4.0 and pH 6.5 showed increased activity of bentonite and MgCl_2_ for removal of CP relative to the pH 7.0, 8.0, or 9.0 extracts, as long as extracts were incubated at 4°C (Figure 2). The pH 6.5 extract in the presence of bentonite and MgCl_2_ reduced TMV CP levels by ~55% (Figure 2), but the pH 4.0 extract in the presence of bentonite and MgCl_2_ reduced CP contamination by ~75%. Therefore, pH 4.0 and incubation at 4°C was used in subsequent purification with bentonite and MgCl_2_, but the most effective concentrations of MgCl_2_ and bentonite remained to be determined.

**Figure 3:**
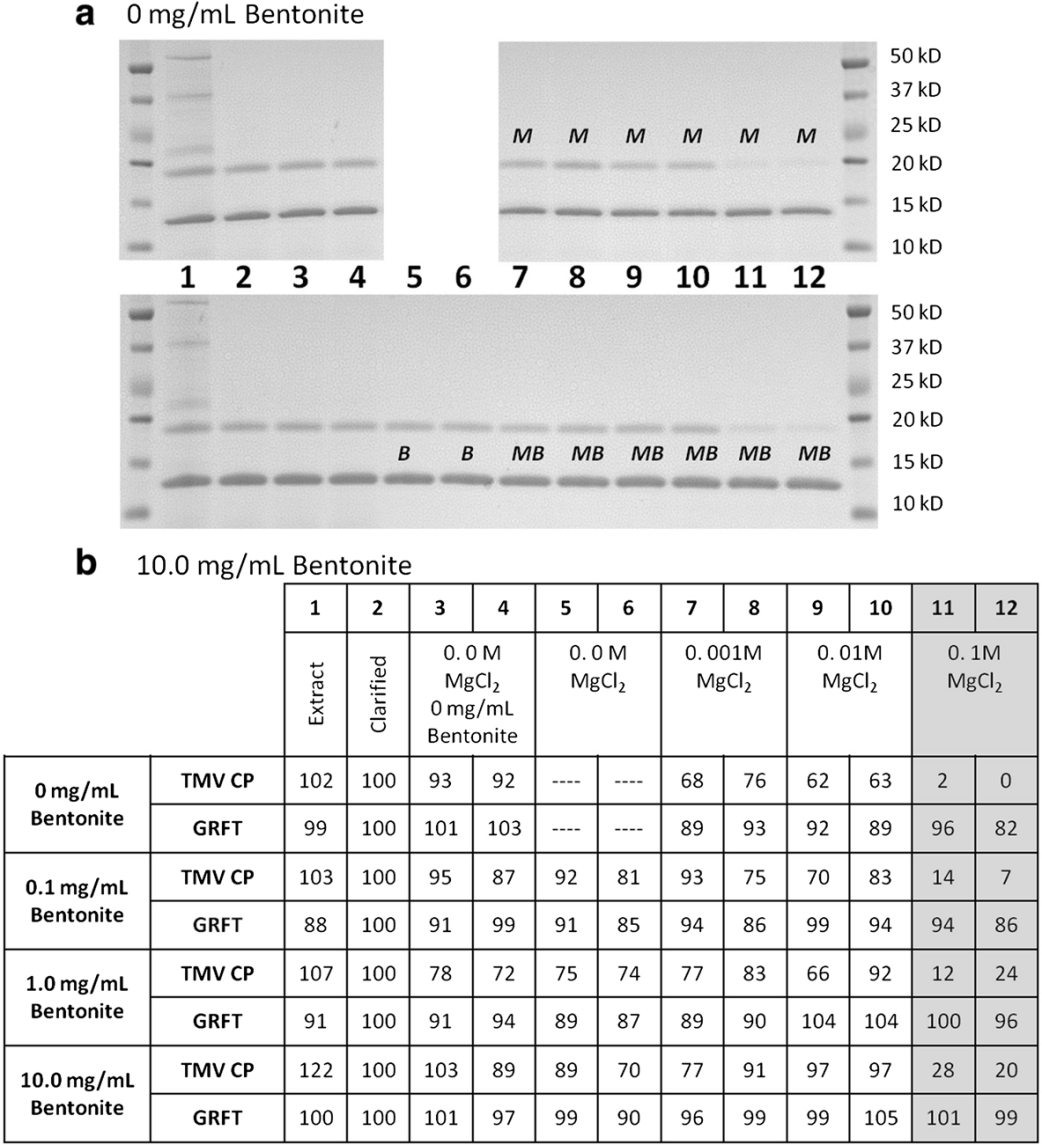
**Effects of MgCl**_**2**_ **(0, 0.001 M, 0.01 M, 0.1 M) on the protein content of GRFT extracts treated with bentonite.** Coomassie stained SDS-PAGE was used to see gross changes in purity of the GRFT extracts in both **a** and **b**. The initial GRFT extract is represented in sample 1 and subsequent heat & centrifugation step at sample 2. The supernatant was treated with multiple MgCl_2_ concentrations (samples 5–12) in duplicate with the corresponding bentonite concentration 0 mg/mL bentonite **(a)** or 10 mg/mL bentonite **(b)**. The resulting supernatants of overnight treatment with MgCl_2_ and/or bentonite are as follows; (3&4) 0 MgCl_2_ & 0 bentonite, (5&6) 0 MgCl_2_, (7&8) 0.001 M MgCl_2_, (9&10) 0.01 M MgCl_2_, (11&12) 0.1 M MgCl_2_. Samples 11 and 12 both show a dramatic reduction in coat protein after treatment with 0.1 M MgCl_2_ irrespective of the bentonite concentration. Table: Densitometry readings of SDS-PAGE represented in **a** and **b** including two additional bentonite concentrations not shown. The shaded area represents an extract condition achieving greater than 80% purity of GRFT.

Multiple concentrations of bentonite and MgCl_2_ were investigated in pH 4.0 buffer to determine the optimal concentration for removal of TMV CP. Bentonite was added to GRFT extracts at a range of 0 mg/mL to 10 mg/mL and MgCl_2_ was added in a range of 0 – 0.1 M with each concentration tested in combination and separately to determine the most effective concentration to aggregate TMV. Figure 3 shows that 0.1 M MgCl_2_ had a similar impact on TMV CP removal at all bentonite concentrations tested, with bentonite having a minimal impact on TMV levels in the absence of MgCl_2_. Removal of 75 – 95% of TMV CP was observed with the addition of 0.1 M MgCl_2_ (Figure 3), but bentonite in the absence of MgCl_2_ only removed 10 – 30% of the CP contaminant. The addition of bentonite and MgCl_2_ at their most effective concentrations, 10 mg/mL and 0.1 M, respectively, failed to be additive in their effects on TMV CP removal. The largest reduction in TMV CP levels was observed in the absence of bentonite with the addition of 0.1 M MgCl_2_, reducing TMV CP contamination by greater than 95% (Figure 3). Bentonite has been proven to help in the clarification process of plant extracts and we continued to explore its impact on purification, but maintain that it has minimal impact on TMV CP removal from pH 4.0 extracts.

**Figure 4:**
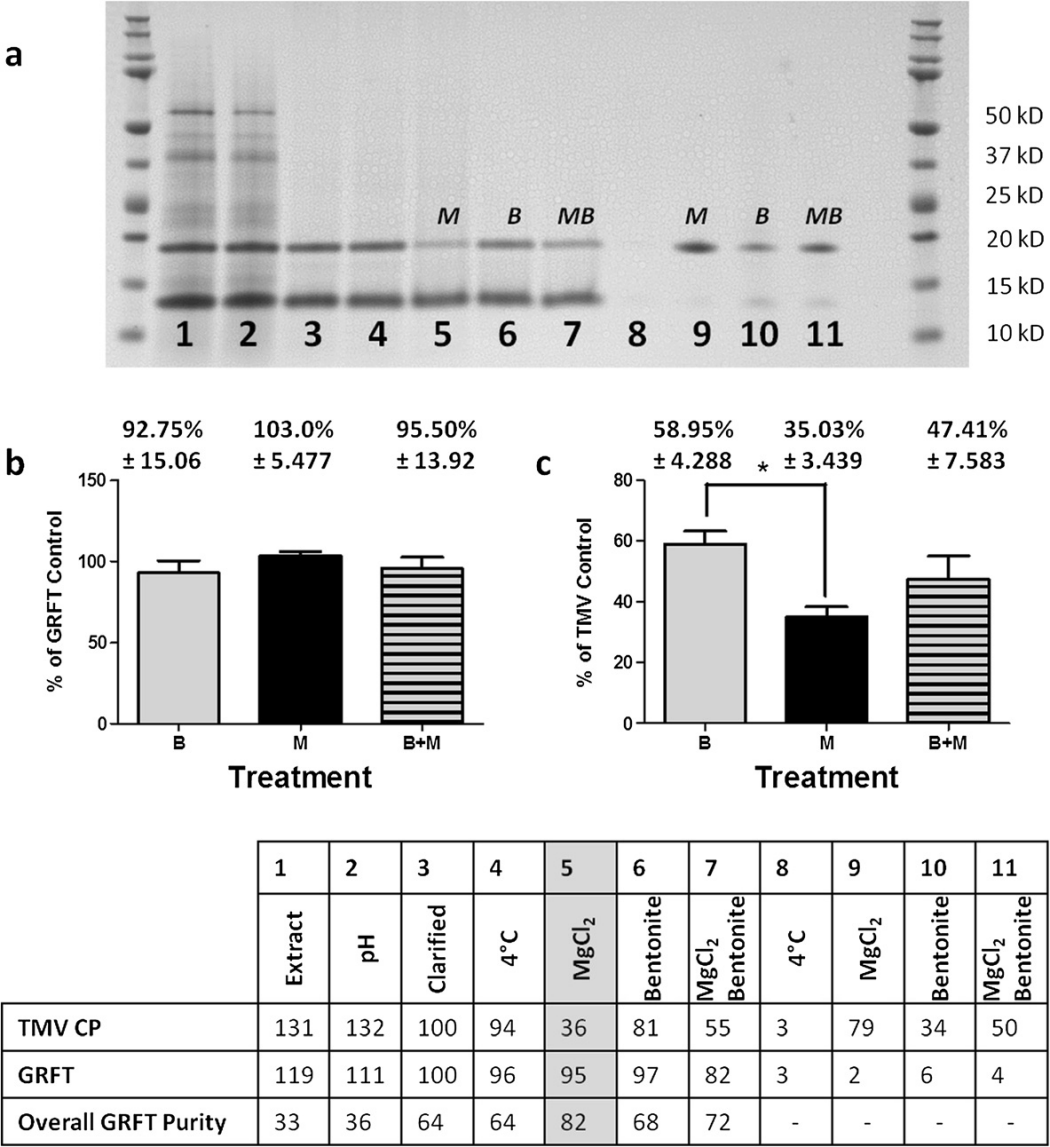
**Summary of the effects of 0.1 M MgCl**_**2**_ **and 10 mg/mL bentonite on GRFT extract purity. a)** Coomassie stained SDS-PAGE showing the initial extraction of GRFT (1), subsequent pH adjustment to pH4 (2) and heating to 55°C followed by centrifugation (3). The resulting supernatant was untreated (control) or treated with MgCl_2_ and/or bentonite and stirred overnight at 4°C. The following samples are the resulting supernatants; (4) Control −4°C, (5) 0.1 M MgCl_2_, (6) 10 mg/mL Bentonite, (7) 0.1 M MgCl_2_ & 10 mg/mL Bentonite . The following samples are the resulting pellets after treatment; (8) Pellet - 4°C, (9) Pellet - 0.1 M MgCl_2_, (10) Pellet - 10 mg/mL Bentonite, (11) Pellet - 0.1 M MgCl_2_ & 10 mg/mL Bentonite. **b)** Comparison of gp-120 binding GRFT concentrations relative to the control sample (4). Determined through a gp-120 binding ELISA and represented as a percentage of functional GRFT in the control extract. **c)** Comparison of the TMV coat protein concentration relative to the control extract. Determined through densitometry measures of TMV specific westerns and represented as a percentage of TMV in the control extract. b and c both analyzed by a one-way ANOVA with Bonferroni’s multiple comparison test comparing all groups. * represents a post-hoc test with a p-value < 0.05. Table: Densitometry readings of SDS-PAGE represented in a. The shaded area represents an extract condition achieving greater than 80% purity of GRFT.

Since the goal was to provide an alternative, less expensive, and less complex purification method the steps outlined thus far were implemented on a larger scale with fewer variables explored. GRFT was extracted and aliquoted after temperature treatment. GRFT extract aliquots were untreated (control) or treated with 0.1 M MgCl_2_ and/or 10 mg/mL bentonite and all were incubated overnight at 4°C. Extracts were compared via SDS-PAGE with densitometry and again showed that the greatest reduction of TMV occurred in the 0.1 M MgCl_2_ treatment group (Figure 4A). The pellets correlating to the specific treatments contained large amounts of TMV CP likely through forced aggregation and removal during centrifugation. Minimal loss of functional GRFT is observed in any of the treatment groups when compared via HIV-1 gp120 binding ELISAs (Figure 4B). When western blots targeting TMV CP were quantified by densitometry a reduction of 65% was observed in extracts treated with MgCl_2_, which is significantly decreased compared to the bentonite-only treated extract (Figure 4C). The table in Figure 4 shows GRFT purity in the four extracts ranges from 64 – 82% with the TMV content of extracts treated with MgCl_2_ reduced by as much as 66%.

**Figure 5:**
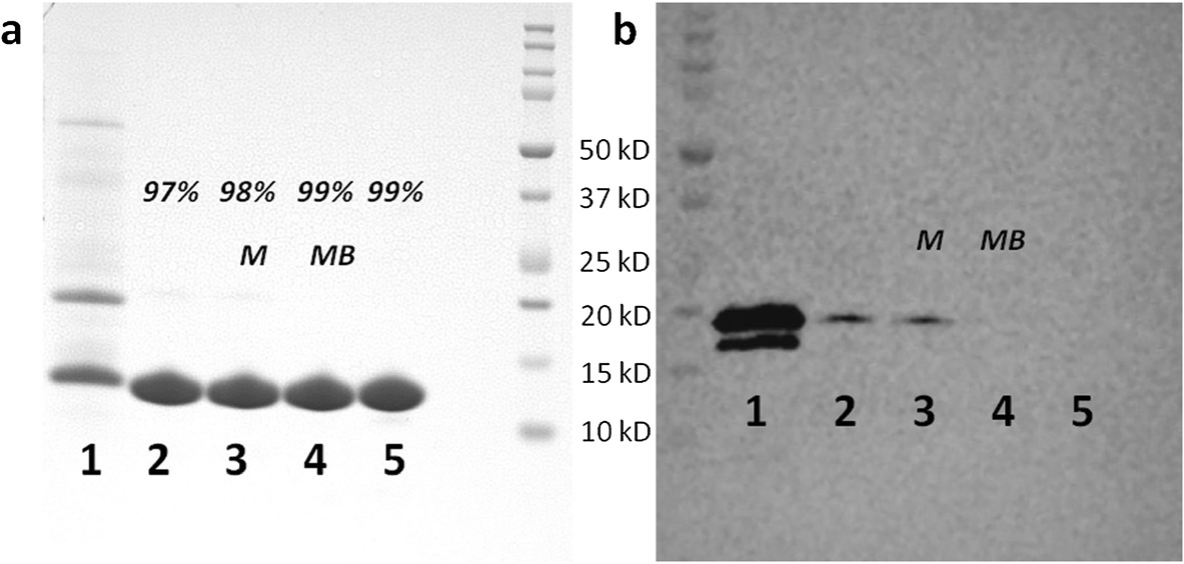
**Purity and TMV CP content of GRFT extracts after Capto** **™** **MMC purification.** SDS –PAGE **(a)** and TMV western blots **(b)** were used to visualize purity and TMV contamination. The initial extract (1) contains both GRFT and TMV CP as well as other plant protein contaminants. Lanes 2–4 were loaded with 15 μg of chromatography purified protein from the extracts that had previously been heated and treated with: control – no treatment (2), 0.1 M MgCl_2_ (3), 0.1 M MgCl_2_ & 10 mg/mL bentonite (4). Included for comparison was 15 μg of GRFT purified by the previously published methodology O’Keefe *et al.,* [4] (5). Above each lane is the densitometry determined GRFT purity.

In an effort to achieve greater than 95% purity of GRFT a chromatography step was explored. Extracts were heated to 55°C and treated with MgCl_2_ or MgCl_2_ and bentonite overnight after which they were filtered through a 0.2 μm filter and loaded onto a Capto® MMC column. The Capto® MMC resin is a multimodal cation exchanger that does not require buffer exchange and was chosen to help minimize purification steps. After chromatography trace amounts of TMV can be seen in SDS gels and western blots in both the untreated-control and the MgCl_2_ treated samples with no TMV contamination observed in the MgCl_2_ and bentonite treated extract (Figure 5). Purity of all extracts after Capto MMC chromatography was greater than 97%, with the bentonite and MgCl_2_ treatment group showing a purity of >99% by SDS-PAGE densitometry. The recovery levels and activity of the purified GRFT were assessed by gp120 binding ELISAs with very good recovery levels achieved in all treatments undergoing MMC chromatography. A minimum of 84.5% was recovered in the MgCl_2_ treated extract after it was purified by chromatography and a maximum recovery of 92.1% was observed in the control extract that was purified by chromatography (Table 1). All extracts maintained an activity level at least 80% of GRFT purified by the previously published method [4], with GRFT purified by MgCl_2_ & bentonite having 100.8 ± 4.33% of the activity (Table 1). In summary, purification of GRFT from *N. benthamiana* by heating to 55°C, overnight treatment with MgCl_2_ & bentonite, and Capto® MMC chromatography recovered 88% of the GRFT with >99% purity and 100% activity of the original purification method.

**Table 1:** **Quantification of activity and recovery of GRFT from the plant extracts**

**Purification method**	**% gp-120 Binding**	**% GRFT Recovery**
Heat and Chromatography	90.19 ± 1.53	92.13 ± 3.29
Heat, MgCl_2_, Chromatography	80.25 ± 5.08	84.54 ± 6.84
Heat, MgCl_2_ & Bentonite, Chromatography	100.8 ± 4.33	87.73 ± 4.82

**Figure 6:**
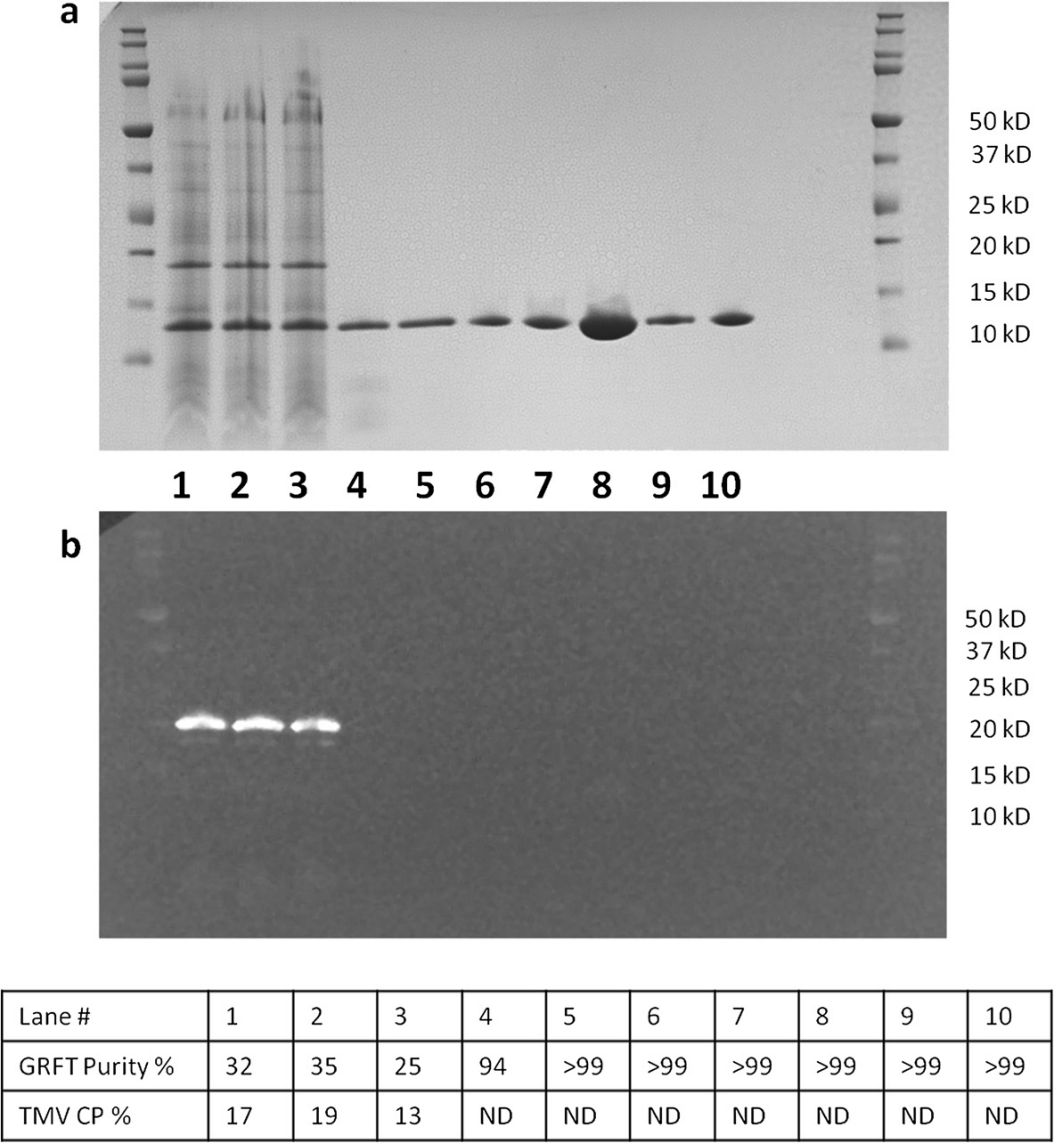
**Analysis of industrial pilot scale purification of GRFT.** Representation of the purity, by SDS –PAGE **a,** and TMV CP content, by western blot **b,** of GRFT during industrial pilot scale purification. Sample volumes loaded on the gel were normalized to the initial extraction volume. The initial extract (1) contains both GRFT and TMV CP as well as other plant protein contaminants. The extract was pH adjusted to pH4 (2)heated (3) and filter pressed(4). After which the extract was incubated overnight with MgCl_2_ and bentonite and simultaneousely filter pressed and sterile filtered (5). Samples 6–7 are the GRFT final product At 1x volume, 2xvolume and 15 μg of protein. Included for comparison was 1.5 μg and 3.0 μg of GRFT purified by the previously published methodology O’Keefe *et al.,* [4] (5). The table represents the densitometric measures of GRFT and TMV protein content in the extracts.

Utilizing the information yielded from small scale experiments GRFT was purified at an industrial pilot scale (>5 kg of plant material) in a GMP ready facility. At this scale it was necessary to replace all centrifugation steps with a filter press because of the impracticality of centrifugation at scale. Ten liters of extraction buffer was added to 5 kg of *N. benthamiana* and the tissue was ground to yield 11.4 L of initial extract containing 2599.73 mg of GRFT for a total of 519.9 mg of GRFT per kg of plant material (Table 2). The extract was pH adjusted, heated, and filter pressed, which removed TMV CP and purified GRFT to >90% (Figure 6). The remaining small protein contaminants were removed by overnight incubation with 10 mg/mL of bentonite and 0.1 M MgCl_2_ and subsequent filter pressing. The extract underwent filter press and sterile filtration simultaneously and at this stage was >99% pure, and sterile with only minimal color. It should be noted that the purification process could be halted at this point depending on the application of the process and necessity for >99% pure colorless product. Capto® MMC chromatography was used to remove the color and simultaneously affect a buffer exchange into phosphate buffered saline pH7.4. Purity was maintained with greater than 99% purity of the final product and recovery of 66–72.25%, determined by densitometry of SDS-PAGE (Figure 6) and gp120 ELISA respectively (Table 2). Because of the GMP-ready nature of the purification scheme, endotoxin levels were assessed and found to be 0.20 EU/mL of final product with a GRFT concentration of 1.20 mg/mL, which should be sufficiently low depending on the dosage and administration route (Table 3). In summary, the purification method developed for GRFT herein provided a >99% pure product with >66% recovery while maintaining low endotoxin levels at industrial pilot scale and could be further scaled without modification to the process.

**Table 2:** **gp120 ELISA assessed GRFT recovery during industrial pilot purification**

**Sample**	**Green juice**	**Filter press #1**	**Filter press #2**	**Eluate**
**GRFT (mg)**	2599.73	1816.34	1576.52	1543.57
**% of GJ**		79%	68%	66%

**Table 3:** **Endotoxin levels in industrial pilot purified GRFT**

	**GRFT Concentration (mg/mL)**	**Endotoxin (EU/mL)**
GRFT Final	1.20	0.204

## Conclusions

GRFT has been described as a promising topical microbicide component with potential to neutralize a broad range of viruses including HIV-1, Herpes simplex virus type 2, Japanese encephalitis virus, Hepatitis C virus, and SARS-CoV [1,2,10-12]. The safety and efficacy profile of GRFT as an HIV entry inhibitor was demonstrated in rabbits, on human cells, and in a sensitive murine HSV-2 susceptibilty assay [2,4,6]. A comprehensive set of highly promising data confirming the potential for use of GRFT in HIV prevention and even treatment, has led to considerable enthusiasm from policy makers and funders in bringing GRFT-based topical microbicides to clinical trial [2,4,6,13-16]. We have been motivated to improve and simplify the GRFT extraction protocol we initially developed [4] in anticipation of future need to manufacture extremely large amounts of this protein, preferably in geographic areas where the HIV-1 epidemic has the most severe population-level impact. The infectious TMV vector system we use to produce GRFT requires only modest investment in plant controlled environment growth facilities, and does not require use of sophisticated *Agrobacterium* infiltration methods that are increasingly used in plant-made pharmaceuticals research. Beyond the additional regulatory concerns associated with *Agrobacterium*-launch vector systems, we have found that unacceptable levels of bacterial endotoxin contaminate GRFT extracts when these are used to express GRFT (unpublished data). Endotoxin removal after *Agroinfiltration* inserts yet another costly (and relatively inefficient) step in protein extraction, making the infectious TMV vectored expression approach more appealing for the extremely cost-sensitive manufacturing of topical microbicide components. Of course, the TMV vectored approach we use does present a downside in the need to efficiently remove the contaminating virus and CP from the extracts. The methodologies we describe in this communication show facile methods for reduction of TMV CP.

We previously produced GRFT in multi-gram quantities in *N. benthamiana*, but the purification methodology used required specialized ceramic filtration devices, two chromatography techniques, ultrafiltration- diafiltration and had relatively low recovery from infected extracts, 30% possibly increasing to 50% [4]. Of the purification techniques we describe here, using a combination of heat (55°C), MgCl_2_ & bentonite, and a single chromatographic step were most effective. This three step approach produced GRFT with no loss in activity or purity, while recovering nearly 90% of the extracted GRFT. Each step targets various contaminating proteins within the plant extract. Heating the extract at 55°C removes the majority of the native plant proteins, which lack the thermostability of GRFT. Exposing the extract to a combination of MgCl_2_ and bentonite removes the TMV CP contaminant, originating from the TMV expression vector. Bentonite and MgCl_2_ work in tandem, with bentonite known to strip coat protein from the TMV RNA and potentially the MgCl_2_ causing either structural shifts in the RNA causing aggregation or cleaving the RNA causing aggregation [9,17,18]. Either mechanism is plausible for the aggregation of TMV in the plant extract. Our data shows the necessity of both MgCl_2_ and bentonite to be present for optimal TMV removal when coupling to a single chromatography step. Utilizing Capto® MMC resin allows for removal of trace protein impurities by binding GRFT to the resin and eluting based upon the isoelectric point of the protein with unobservable amounts of TMV left in extracts treated with bentonite and MgCl_2_. Stripping of the CP from TMV likely reduces its ability to bind Capto® MMC resin and allows for less TMV contaminant binding and co-elution. All of the techniques, heating, cooling, pH adjustment, and chemical additives, used to purify GRFT are all facile, scaleable and amenable to technology transfer to facilities in resource poor countries. In an effort to demonstrate the scaleability and and amenability of technology transfer the process was performed at pilot scale in a GMP ready industrial facility with only slight reductions in the recovery, while providing pure active GRFT with low endotoxin levels. The methodology maintains increased recovery at scale, reducing per unit costs of GRFT, which should increase the availability of GRFT for HIV entry-inhibition in resource limited settings.

The techniques discussed here can be applied to other recombinant protein production methods to address similar protein contaminants such as native plant proteins, RuBisCO, and TMV CP. Recombinant proteins purified from transgenic or transiently expressing plants both can utilize portions of the methodology to remove contaminating proteins. It is possible that the manufacturing process could be further abbreviated with the use of transgenically expressed GRFT.

In conclusion, purification of GRFT at scale using this technique should provide multi-kilogram quantities of GRFT at a low cost with minimal product loss suitable for practical development of GRFT-based topical microbicides.

## Methods

### GRFT expression

GRFT was expressed in *N. benthamiana* using the same plasmid and methodology described in O’Keefe et al., [4]. Briefly, a TMV-based expression vector system containing the GRFT amino acid sequence was used to generate infectious RNA transcripts. Infectious transcripts were inoculated on *N. benthamiana* (~24 days post sowing) by gentle abrasion.

### GRFT extraction – leaf punches

Ten days after inoculation, 8 mm leaf punches were randomly taken from eight *N. benthamiana* plants. Two leaf punches were homogenized in 200 μL of each extraction buffer. Extraction buffers ranged from pH 2–10 consisting of 100 mM buffering agent (glycine, sodium acetate, Tris, sodium phosphate) with and without 100 mM sodium chloride. Samples were centrifuged 15,000 × g for 15 minutes and the supernatant removed. The supernatants were then treated with the application of 60°C, 70°C, or 80°C for ten minutes and compared to an ambient temperature control sample for each buffer.

### GRFT extraction – bench scale

Ten days after inoculation, leaf tissue was harvested and homogenized in the appropriate extraction buffer with a ratio of 1:2 (Plant tissue (g): Extraction buffer (mL)). Extraction buffer composition was either 100 mM sodium acetate, 300 mM sodium chloride, 20 mM ascorbic acid, 10 mM sodium metabisulfite for buffers with a pH range of 4.0 - 6.5 and 100 mM tris-hydrochloride, 300 mM sodium chloride, 20 mM ascorbic acid, 10 mM sodium metabisulfite for buffers in the pH range of 7.0 -9.0. Extract material was filtered through 4 layers of cheesecloth and 1 layer of miracloth, heated to the desired temperature for 15 minutes, and centrifuged at 15,000 × g for 15 minutes. In some experiments the purification process was halted at this point and the extract tested for purity by SDS PAGE, but in an effort to remove the TMV coat protein additional alterations were made to the extract. The semi-pure extract was treated with 0 – 0.1 M MgCl_2_ and 0 – 10 mg/mL bentonite at varying pHs and then incubated at varying temperatures over night to determine if any combination of bentonite, MgCl_2_, pH, and temperature could help remove the coat protein. After 16 hrs at their specified temperature all extracts were centrifuged at 15, 000 × g for 15 minutes and the supernatant removed. Pellets were resuspended in equivalent volumes of 2× SDS running buffer. Pellet and supernatant samples were analyzed by SDS-PAGE.

### SDS-PAGE with densitometry

Sample buffer (4 X SDS) containing BME was added to all samples and samples were then boiled at 95°C for 10 minutes. Twenty microliters of sample was loaded on 12% Bis-Tris gels with the addition of 5 μL of protein ladder to the outer wells. Gels were ran at 200 V for 37 minutes then stained with coomassie stain for 1 hour and destained for 3 hours. Gels were imaged on a Kodak Image station 4000R Pro using Carestream SE M software. The software was then used to mark the lanes on the gell and find resulting bands. Bands were analyzed based on intensity and size. GRFT purity of each sample was determined based on the band sizes and strength of contaminating proteins in the same lane. The percentage of TMV CP or GRFT for each purification step was relative to the sample representing the step preceding the parameters that were investigated. Therefore all GRFT and TMV CP samples are represented as a percentage of the unmodified control.

### GRFT gp120 ELISA

In a 96-well plate, wells were coated with 200 μL of 1 ng/mL gp120 (Protein Sciences, Meriden, CT) in PBS and incubated overnight at 4°C. Plates were washed with PBS-T and blocked with 5% PBS-T-M for 2 hours at room temperature. Wells were washed with PBS-T and incubated at room temperature for 2 hours with sample diluted 1:10 in PBS and further diluted in series 1:5 down the plate. Purified GRFT, uninfected plant extract, PBS sample and no gp120 coating controls were included in all ELISAs. Plates were washed and incubated for 1 hour at room temperature with RB anti-GRFT serum dilute 1:10,000 in 1% PBS-T-M. After washing the plate, the secondary antibody, GT anti-rabbit conjugated to HRP, was added to each well diluted 1:20,000 in 1% PBS-T-M. Plates were again washed and the ultra sensitive TMB substrate added to each well. After 3 minutes 0.2 M H2SO4 was added to stop the reaction and the colorimetric change was read at OD 405 nm on a Beckman- Coulter plate reader. The collected data was background subtracted and analyzed for the relative change in GRFT concentration between purification methods.

### TMV western blot

Samples were ran on SDS-PAGE via methodology described earlier and then transferred to a PVDF membrane via BioRad transblot turbo system. Membranes were incubated for 1 hour with 3% PBS-T-M (phosphate bufferd saline with 0.5% Tween20 and 3% non-fat dry milk) at room temperature, washed with PBS-T three times and incubated overnight at4°C with rabbit antibody against TMV 1:5000 in 3% PBS-T-M. Blots were then washed with 3% PBS-T and incubated with goat anti-rabbit HRP conjugated antibody diluted 1:10,000 in 3% PBS-T-M for 1 hour at room temperature. Blots were washed and developed with ECL Prime development kit (GE Lifesciences). Blots were exposed on a Kodak Image Station 4000 R Pro using Carestream SE M software. Software was then used to identify and analyze visible bands based on intensity and size.

### Chromatography and purity verification

GRFT extract was purified using an AKTA Purifier with a XK16 column packed with 10mLs of Capto™ MMC resin (GE Life Sciences). After overnight incubation the semi-pure extract was filtered through a 0.2 μm filter and loaded on to the column at a rate of 5 mL/minute. A two-step gradient of 90% and 100% phosphate buffered saline (137 mM NaCl, 2.7 mM KCl, 10 mM NaH_2_PO_4_, 2 mM KH_2_PO_4_) pH7.4 was used to elute GRFT from the column. Fractions (10 mL) were collected and pooled after being checked for the presence of GRFT through SDS-PAGE. The purity of the concentrated GRFT was verified by an overloaded SDS-PAGE and a western blot against the TMV CP. The glycan binding capabilities of the purified GRFT were determined through gp120 binding. The purified extract was compared to GRFT purified with the previously published methodology in over-loaded SDS-PAGE, western blots against TMV and gp120 ELISAs.

### Industrial pilot scale purification

Five days after infectious transcript inoculation, plants were harvested and the rTMV was isolated. The isolated rTMV was used to inoculate approximately 600 plants through high velocity spraying with diatomaceous earth in a 100 mM phosphate buffer pH 7.4. Ten days after spray inoculation, five kg of leaf tissue was harvested and homogenized in extraction buffer (100 mM sodium acetate, 300 mM sodium chloride, 20 mM ascorbic acid, 10 mM sodium metabisulfite pH4.0) with a ratio of 1:2 (Plant tissue (g): Extraction buffer (mL)). The extract pH was adjusted back to pH 4.0 and heated to 55°C for 15 minutes. The heated extract was immediately filter pressed through a 0.3 μm filter (Ertel Alsop, M-853) with the addition of the filter aid Cell Pure® (30 g/L). The extract was incubated overnight in a holding tank with continuous stirring at 4°C with the addition of 10 mg/mL of bentonite and 0.1 M MgCl_2_. After overnight incubation the extract was filter pressed again through a 0.3 μm filter and an inline sterilization filter (Sartorius, Sartopore 2). Capto® MMC chromatography was performed as described above, but the resin was packed in a sterilized AxiChrom 50/300 column. Purity was assessed via previously discussed methodology of gp120 ELISA, SDS-PAGE with densitometry, and TMV western blot with densitometry. Additionally, endotoxin levels were assessed with the methodology explained below.

### Endotoxin analysis

Contaminating endotoxin levels in the final GRFT product purified at industrial pilot scale was analyzed with Charles River Laboratory Endosafe® PTS. Twenty-five μL of GRFT solution was added to each of the four wells on a Limulus Amebocyte Lysate Test cartridge and the endotoxin levels were analyzed accordingly.

### Statistical analysis

All statistical analysis and summary graphical representations were performed on Graph Pad Prism® 5.0.

## References

[1] Mori T, O’Keefe BR, Sowder RC, Bringans S, Gardella R, Berg S (2005). Isolation and characterization of griffithsin, a novel HIV-inactivating protein, from the red alga Griffithsia sp. J Biol Chem.

[2] Nixon B, Stefanidou M, Mesquita PM, Fakioglu E, Segarra T, Rohan L (2013). Griffithsin Protects Mice from Genital Herpes by Preventing Cell-to-Cell Spread. J Virol.

[3] Emau P, Tian B, O’Keefe BR, Mori T, McMahon JB, Palmer KE (2007). Griffithsin, a potent HIV entry inhibitor, is an excellent candidate for anti-HIV microbicide. J Med Primatol.

[4] O’Keefe BR, Vojdani F, Buffa V, Shattock RJ, Montefiori DC, Bakke J (2009). Scaleable manufacture of HIV-1 entry inhibitor griffithsin and validation of its safety and efficacy as a topical microbicide component. Proc Natl Acad Sci U S A.

[5] Giomarelli B, Schumacher KM, Taylor TE, Sowder RC, Hartley JL, McMahon JB (2006). Recombinant production of anti-HIV protein, griffithsin, by auto-induction in a fermentor culture. Protein Expr Purif.

[6] Kouokam JC, Huskens D, Schols D, Johannemann A, Riedell SK, Walter W (2011). Investigation of griffithsin’s interactions with human cells confirms its outstanding safety and efficacy profile as a microbicide candidate. PLoS One.

[7] Moulaei T, Shenoy SR, Giomarelli B, Thomas C, McMahon JB, Dauter Z (2010). Monomerization of viral entry inhibitor griffithsin elucidates the relationship between multivalent binding to carbohydrates and anti-HIV activity. Structure.

[8] Ziolkowska NE, Shenoy SR, O’Keefe BR, McMahon JB, Palmer KE, Dwek RA (2007). Crystallographic, thermodynamic, and molecular modeling studies of the mode of binding of oligosaccharides to the potent antiviral protein griffithsin. Proteins.

[9] Brakke MK, Van Pelt N (1969). Influence of bentonite, magnesium, and polyamines on degradation and aggregation of tobacco mosaic virus. Virology.

[10] O’Keefe BR, Giomarelli B, Barnard DL, Shenoy SR, Chan PK, McMahon JB (2010). Broad-spectrum in vitro activity and in vivo efficacy of the antiviral protein griffithsin against emerging viruses of the family Coronaviridae. J Virol.

[11] Takebe Y, Saucedo CJ, Lund G, Uenishi R, Hase S, Tsuchiura T (2013). Antiviral Lectins from Red and Blue-Green Algae Show Potent In Vitro and In Vivo Activity against Hepatitis C Virus. PLoS One.

[12] Ishag HZ, Li C, Huang L, Sun MX, Wang F, Ni B (2013). Griffithsin inhibits Japanese encephalitis virus infection in vitro and in vivo. Arch Virol.

[13] Hoorelbeke B, Xue J, Liwang PJ, Balzarini J (2013). Role of the Carbohydrate-Binding Sites of Griffithsin in the Prevention of DC-SIGN-Mediated Capture and Transmission of HIV-1. PLoS One.

[14] Meuleman P, Albecka A, Belouzard S, Vercauteren K, Verhoye L, Wychowski C (2011). Griffithsin has antiviral activity against hepatitis C virus. Antimicrob Agents Chemother.

[15] Xue J, Gao Y, Hoorelbeke B, Kagiampakis I, Zhao B, Demeler B (2012). The role of individual carbohydrate-binding sites in the function of the potent anti-HIV lectin griffithsin. Mol Pharm.

[16] Barton C, Kouokam JC, Lasnik AB, Foreman O, Cambon A, Brock G (2014). Activity of and effect of subcutaneous treatment with the broad-spectrum antiviral lectin griffithsin in two laboratory rodent models. Antimicrob Agents Chemother.

[17] Sherwood JL, Fulton RW (1982). The specific involvement of coat protein in tobacco mosaic virus cross protection. Virology.

[18] Powell CA (1975). The effect of cations on the alkaline dissociation of tobacco mosaic virus. Virology.

